# The metabolic effect of combined liraglutide treatment and lifestyle modification on obese adolescents in a tertiary center, Riyadh

**DOI:** 10.3389/fendo.2025.1573109

**Published:** 2025-04-15

**Authors:** Amir Babiker, Haifa Alfaraidi, Gadah Aljarallah, Joud Albaraki, Reem Alharbi, Nouf Alsomali, Abeer Alkhalaf, Nagarajkumar Yenugadhati, Fahad Al Juraibah, Ibrahim Al Alwan

**Affiliations:** ^1^ College of Medicine, King Saud Bin Abdulaziz University for Health Sciences, Riyadh, Saudi Arabia; ^2^ King Abdullah Specialized Children’s Hospital, King Abdulaziz Medical City, Ministry of National Guard Health Affairs, Riyadh, Saudi Arabia; ^3^ King Abdullah International Medical Research Centre (KAIMRC), Ministry of National Guard Health Affairs, Riyadh, Saudi Arabia; ^4^ College of Public Health and Health Informatics, King Saud Bin Abdulaziz University for Health Sciences, Riyadh, Saudi Arabia

**Keywords:** adolescents, BMI, hemoglobin A1c, metabolic, lifestyle, liraglutide, obesity, Saudi Arabia

## Abstract

**Background:**

Obesity has increased in adolescents with a rising incidence of metabolic consequences, including type 2 diabetes, necessitating new pharmacotherapeutic approaches. Liraglutide is the first FDA-approved therapy for obesity in adolescents in less than a decade. We assessed its efficacy combined with lifestyle interventions in our patients.

**Methods:**

A retrospective cohort study was conducted at a specialized children hospital in Riyadh (2019–2022). All patients had simple, non-syndromic obesity and received intensive education on lifestyle modification. Data was collected from patients in two groups: Lifestyle modification (LifeSG) Vs Lifestyle and Liraglutide (LiraglG). Comparisons of two repeated measures obtained at T1 (baseline) and T2 (6-9 months) or T3 (9-12 months), including changes in body mass index (BMI), glycated hemoglobin (HbA1c) and other metabolic markers, were performed in the two matched groups using paired t-tests. Regression analysis using linear mixed models (SAS 9.4) were used to assess the effect of treatment status over time (P-value ≤ 0.05).

**Results:**

Data collected from 138 patients (n=69 in each group) with mean BMI and HbA1c of 35.78 kg/m^2^ and 5.85%, respectively. Notably, BMI declined by 0.48 kg/m^2^ over time in the LiraglG (p=0.003). An interaction effect (p=0.027) suggested a treatment impact until the first follow-up, which was not sustained thereafter. LifeSG exhibited no significant changes in biomarkers throughout T1-T3 period. In contrast, significant reductions were observed in BMI between T1-T2 (p=0.0057) and T1-T3 (p=0.010), total cholesterol (T1-T2) (p=0.023), alkaline phosphatase (T1-T3) (p<0.05) and low-density lipoprotein mean levels (T1-T3) (p=0.05) in the LiraglG group. A decline of 0.13% in A1c was observed in LiraglG; which may not clinically meaningful except in patients with pre-diabetes range of A1c (≥ 5.8%).

**Conclusion:**

Liraglutide combined with lifestyle intervention is effective in treating obese Saudi adolescents, especially in the first 6-9 months. Continuous lifestyle intervention plays a key role in sustainability.

## Introduction

Childhood obesity is defined as having a body mass index (BMI) at or above the 95th percentile of the growth reference median for age (1). In recent years, obesity has become a significant global issue, resulting in a pandemic (2). The prevalence of childhood and adolescent obesity has been increasing worldwide, with Saudi Arabia being no exception. Approximately one-fifth of Saudi children and adolescents are either overweight or obese, with 11.4% classified overweight and 9.4% being obese (3). Many factors are associated with obesity in Saudi children, including age, higher family income, and mother’s level of education (4, 5).

Managing pediatric obesity can be challenging, and adherence to lifestyle interventions, such as healthy diet and maintaining regular exercise, can be difficult. Therefore, it is essential to identify interventions that can successfully treat obesity in both the short- and long-term periods, especially given the detrimental effect of obesity on the cardiometabolic health of children and adolescents, including the risk of developing type 2 diabetes.

Recent clinical guideline endorsed by the American Academy of Pediatrics suggested that physicians should offer anti-obesity medication to youth struggling with obesity (6). Glucagon-like peptide-1 (GLP-1) receptor agonists are anti-obesity medication that enhance satiety through their action on the central nervous system and delay gastric emptying. Numerous randomized clinical trials comparing various GLP-1 receptor agonists, such as liraglutide and semaglutide, to placebo in overweight or obese adults have demonstrated that GLP-1 receptor agonists resulted in more significant weight loss and improvement in cardiometabolic markers compared to placebo (7–10).

Liraglutide is the first GLP-1 receptor agonist approved by the FDA for weight reduction in obese adolescents over 12 years of age (11). In a randomized, double-blinded clinical trial in obese adolescents, liraglutide resulted in a statistically significant reduction in the standard deviation score of BMI, when compared to placebo (12). A recent meta-analysis that included randomized clinical trials studying the effect of GLP-1 receptor agonists on obese children and adolescents concluded that GLP-1 agonists had a modest effect in improving weight, BMI, HbA1c and systolic blood pressure, with nausea being the most frequent side effect (13).

Local studies documenting the effect of GLP-1 receptor agonists on obese Saudi adolescents are limited. Our study aims to evaluate the efficacy of liraglutide treatment as an adjunct to lifestyle interventions for weight loss and improvement in various cardiometabolic markers among Saudi adolescents treated for obesity at a tertiary pediatric center in Riyadh.

## Materials and methods

A retrospective cohort study was conducted at King Abdullah Specialized Children’s Hospital (KASCH), Riyadh, Saudi Arabia. We included all children and adolescents, aged between 12 and 14 years with simple obesity who met the inclusion and exclusion criteria and were evaluated in the pediatric endocrinology clinics from January 2019 to December 2022. These children were either treated with combined lifestyle modification and liraglutide therapy (LiraglG) or lifestyle modification alone (lifeSG). Those with syndromic obesity or on glucocorticoids were excluded, as were children under 12 years treated “off label” with liraglutide. Children in the LifeSG group were matched for age, gender and BMI with those in the LiraglG.

Patients in both of the study groups received intensive education on lifestyle modification using a 5 As approach (ask, assess, advise, agree and assist) adapted for obesity counseling at our centre. First, we “ask” permission to discuss weight with the adolescent; we remain nonjudgmental and explore the patient’s readiness for change. This is usually accompanied by a thorough “assessment” of dietary and exercise history, followed by comprehensive counselling that includes “advices” on healthy and individualized meal plans set by our specialized dietician. A comprehensive plan including diet, 30-60 minutes of daily moderate-to-high intensity exercise, and behavioral adjustment therapy (including eating habits and adequate sleep), is discussed and “agreed” upon with children and parents or other caregivers. Patients are “assisted” and coached to achieve their individual targets by various members of a multidisciplinary team (MDT) including physicians, dietician, physiotherapist, psychologist and other specialists such as orthopedic and bariatric surgeons. The patient and parents are part of this team and are typically involved in decision-making regarding their choices and preferences. Although, we do not as yet have a set-up of one regular MDT clinic that gathers all of these members caring of children with obesity, the team members maintain regular communication about these patients, and one of them reviews the child at least every 1-2 months in their clinics, if not more frequently during initial consultations.

Data was collected from patients in the two groups by reviewing their electronic medical records. Measures were obtained at baseline (T1), at 6-9 months (T2), and at 9-12 months (T3). This included height, weight, BMI, HbA1c, and metabolic markers for lipids (i.e., total cholesterol, triglycerides, LDL- Low Density Lipoprotein, HDL- High Density Lipoprotein) and liver function (i.e., ALP-Alkaline Phosphatase, ALT-Alanine Transaminase, AST-Aspartate Transferase).

All analyses were performed using SAS 9.4. Descriptive data were reported as mean and standard deviation for continuous variables, and frequencies and percentages for categorical variables. Comparison of two repeated measures were performed using paired t-tests between measurements obtained at baseline (T1) and at two follow-ups (T2 and T3). Linear mixed models were used to perform regression analysis to determine the effect of follow-up (i.e., time) on patient characteristics measured at T1, T2 and T3, and the effect of treatment status (liraglutide and lifestyle changes vs lifestyle alone), including the effect of treatment over time (modeled as an interaction between time and treatment). The random effect of the subjects was modeled as a random intercept in the analysis. Simple effects, i.e., the change in levels of biomarker (or other patient measures) over time, were reported for the liraglutide group where appropriate. A significance level of 0.05 was used to determine statistical significance in all the analyses.

## Results

A total of 138 patients were included (n=69 in each group), matched for age, gender, and BMI (+/-5 kg/m^2^). The distribution of male and female children among treatment groups was similar with 34 girls and 35 boys in each group. In addition to our analysis of data for the whole cohort, we provided further analysis of gender subgroups, with only minor differences observed between genders; most significant differences between the LiraglG and LifSG groups were attributed to changes in the female subgroup. Approximately 17 non-genetic/non-syndromic patients with obesity were excluded as they had initially started on liraglutide but discontinued treatment due to various reasons, including gastrointestinal side effects.

Descriptive data on various patient characteristics and biomarkers at baseline (T1) are provided in **Table 1**, including subgroup analysis by gender. The mean measures for the whole cohort for BMI and A1C were 35.78 kg/m^2^ and 5.85%, respectively. The paired comparisons among the three repeated measures within each treatment group are reported in **Table 2**, showing minor differences between gender subgroups. In the LiraglG, the average measures of the biomarkers evaluated significantly differed at each of the follow-up times. In the liraglutide group, the average BMI at baseline (T1) significantly differed from follow-up measures at T2 and T3. The mean change of BMI from the start of liraglutide to the end of the observation period was 0.95 kg/m^2^, representing approximately a 2.5% reduction in BMI (p=0.01). In the period T1-T3, 24.6% (n=17/69) of patients achieved at least a 5% reduction in BMI, and 14.5% (n=10/69) experienced a 5-10% reduction in weight in the LiraglG group, compared to 15.9% (n=11/69) of patients in the LifeSG group who achieved at least a 5% reduction in their BMI and weight (**Tables 3**, **4**) .Similarly , average total cholesterol level measured at T3 differed significantly from T1 and significant changes in the average LDL levels were observed between T1 and T2, and T1 and T3.

**Table 1:** Baseline characteristics for patients in the lifestyle and liraglutide groups (at T1).

	Lifestyle (n=69)		Liraglutide (n=69)		
Variable	Mean	SD	Mean	SD	P-value*
Age	13.39	0.91	13.39	0.91	1.000
Weight SDS	2.23	0.79	2.26	0.88	0.825
Height SDS	-0.46	1.42	-0.21	1.23	0.299
BMI	34.50	5.26	36.99	5.62	0.011
BMI z-score	2.20	0.53	2.32	0.42	0.164
HA1c	5.79	1.50	5.90	1.15	0.724
Total_Cholesterol	3.98	0.68	4.35	1.02	0.091
Triglycerides	1.26	0.69	1.31	0.96	0.814
LDL	2.54	0.87	2.72	0.86	0.402
HDL	1.05	0.21	1.13	0.22	0.158
ALT	32.47	46.05	26.14	18.39	0.492
AST	30.31	46.96	21.10	7.99	0.302
ALP	200.85	85.14	212.55	100.91	0.622

Female Only					
	Lifestyle (n=34)		Liraglutide (n=34)		
Variable	Mean	SD	Mean	SD	P-value*
Age	13.32	0.94	13.32	0.94	1.000
Weight SDS	2.14	0.66	2.30	0.61	0.825
Height SDS	-0.56	1.58	-0.09	1.10	0.299
BMI	34.39	5.24	36.99	5.78	0.075
BMI SDS	2.13	0.50	2.31	0.47	0.164
HA1c	5.39	0.44	5.76	0.90	0.724
Total_Cholesterol	3.92	0.64	4.06	0.77	0.091
Triglycerides	1.38	0.85	1.25	0.70	0.814
LDL	2.34	0.84	2.67	0.68	0.402
HDL	1.03	0.21	1.11	0.18	0.158
ALT	19.06	6.23	22.00	9.25	0.492
AST	18.56	3.97	18.00	6.10	0.302
ALP	163.88	81.26	179.36	93.03	0.622

Male Only					
	Lifestyle (n=35)		Liraglutide (n=35)		
Variable	Mean	SD	Mean	SD	P-value*
Age	13.46	0.89	13.46	0.89	1.000
Weight SDS	2.32	0.89	2.23	1.07	0.723
Height SDS	-0.37	1.30	-0.33	1.35	0.908
BMI	34.59	5.36	36.99	5.57	0.075
BMI SDS	2.26	0.55	2.32	0.38	0.583
HA1c	6.09	1.94	6.01	1.33	0.872
Total Cholesterol	4.05	0.74	4.57	1.15	0.127
Triglycerides	1.12	0.39	1.37	1.16	0.466
LDL	2.71	0.89	2.78	1.03	0.848
HDL	1.08	0.22	1.16	0.26	0.369
ALT	45.88	62.94	28.67	22.11	0.284
AST	42.06	65.17	22.99	8.55	0.227
ALP	235.65	75.18	232.83	102.63	0.927

*P-value based on independent t-test.

LP, Alkaline phosphatase; ALT, Alanine transaminase; AST, Aspartate transferase; BMI, Body mass index; HbA1c, Glycated hemoglobin; HDL, High density lipoprotein; Kg, Kilogram; LDL, Low density lipoprotein; N, Number; SD, Standard deviation.

**Table 2:** Comparisons between mean differences of cardiometabolic biomarkers in the lifestyle and liraglutide groups at different visits (T1- T3).

Lifestyle										Liraglutide								
Variable	T1_Mean	T1_SD	T2_Mean	T2_SD	T3_Mean	T3_SD	P-value*			T1_Mean	T1_SD	T2_Mean	T2_SD	T3_Mean	T3_SD	P-value*		
							T1-T2	T1-T3	T2-T3							T1-T2	T1-T3	T2-T3
BMI	34.50	5.26	35.38	6.09	34.36	6.13	0.275	0.502	0.470	36.99	5.62	36.21	5.99	35.89	5.70	**0.005**	**0.018**	0.868
HA1c	5.79	1.50	5.68	1.13	6.15	1.90	0.230	0.606	0.203	5.90	1.15	5.98	1.59	5.92	1.76	**0.022**	**0.012**	0.280
Total cholesterol	3.98	0.68	4.05	0.72	4.12	0.72	0.897	0.713	0.586	4.35	1.02	4.31	0.79	4.10	0.95	0.085	**0.024**	0.952
Triglycerides	1.26	0.69	1.23	0.72	1.36	0.88	0.952	0.255	0.316	1.31	0.96	1.10	0.51	1.18	0.75	0.150	0.453	0.226
LDL	2.54	0.87	2.71	0.78	2.75	0.71	0.265	0.359	0.130	2.72	0.86	2.85	0.74	2.81	0.87	0.052	0.059	0.827
HDL	1.05	0.21	1.03	0.20	1.01	0.16	0.672	0.193	0.918	1.13	0.22	1.12	0.21	1.15	0.22	0.804	0.933	0.860
ALT	32.47	46.05	23.78	19.21	25.40	17.55	0.054	0.086	0.121	26.14	18.39	20.82	20.88	19.25	12.82	0.358	0.086	0.282
AST	30.31	46.96	21.80	9.43	21.71	8.18	0.088	0.195	0.540	21.10	7.99	20.68	14.05	19.08	6.23	0.853	0.142	0.337
ALP	200.85	85.14	195.30	75.22	172.15	61.85	0.157	0.236	0.582	212.55	100.91	175.85	87.40	169.66	68.19	0.107	**0.026**	0.478

Variable	T1_Mean	T1_SD	T2_Mean	T2_SD	T3_Mean	T3_SD				T1_Mean	T1_SD	T2_Mean	T2_SD	T3_Mean	T3_SD	P-value*		
BMI	34.39	5.24	35.57	5.65	35.92	5.67	0.672	0.808	0.482	36.99	5.78	36.06	5.92	35.4	5.63	0.250	0.016	0.296
HA1c	5.39	0.44	5.43	0.76	5.95	1.78	**0.013**	0.182	0.289	5.76	0.90	5.96	1.86	5.94	1.97	0.061	**0.049**	0.098
Total cholesterol	3.92	0.64	4.07	0.70	4.02	0.71	0.475	0.131	0.767	4.06	0.77	4.37	0.85	4.25	0.77	0.283	0.361	0.567
Triglycerides	1.38	0.85	1.33	0.84	1.38	1.09	0.376	0.339	0.456	1.25	0.70	0.94	0.38	1.06	0.71	0.529	0.259	0.534
LDL	2.34	0.84	2.77	0.77	2.70	0.58	0.091	0.121	0.401	2.67	0.68	2.89	0.84	2.91	0.85	0.885	0.578	0.830
HDL	1.03	0.21	1.02	0.17	1.02	0.17	0.664	0.067	0.402	1.11	0.18	1.12	0.23	1.21	0.23	0.779	1.000	0.854
ALT	19.06	6.23	17.06	6.28	20.00	13.37	0.467	0.720	0.785	22.00	9.25	15.28	7.61	16.10	10.22	0.122	0.218	1.000
AST	18.56	3.97	18.28	5.09	21.11	8.39	**0.016**	0.713	0.481	18.00	6.10	15.61	4.38	16.90	5.23	0.313	0.066	0.896
ALP	163.88	81.26	162.89	65.54	126.56	46.56	0.149	0.181	0.092	179.36	93.03	130.18	53.38	121.63	40.04	**0.035**	0.072	0.295

Variable	T1_Mean	T1_SD	T2_Mean	T2_SD	T3_Mean	T3_SD				T1_Mean	T1_SD	T2_Mean	T2_SD	T3_Mean	T3_SD	P-value*		
BMI	35.59	5.36	35.22	6.54	32.73	6.27	0.313	0.394	0.228	36.99	5.57	36.36	6.15	36.34	5.81	0.005	0.311	0.297
HA1c	6.09	1.94	5.90	1.36	6.33	2.04	0.462	0.737	0.529	6.01	1.33	6.00	1.24	5.90	1.53	0.215	0.115	0.599
Total Cholesterol	4.05	0.74	4.03	0.76	4.20	0.74	0.369	0.325	0.657	4.57	1.15	4.27	0.76	3.97	1.09	0.036	0.040	0.358
Triglycerides	1.12	0.39	1.14	0.58	1.34	0.68	0.524	0.616	0.547	1.37	1.16	1.23	0.57	1.27	0.77	0.219	0.827	0.317
LDL	2.71	0.89	2.66	0.81	2.78	0.82	0.487	0.118	0.229	2.78	1.03	2.81	0.63	2.71	0.89	0.010	0.056	0.435
HDL	1.08	0.22	1.04	0.23	1.00	0.16	0.408	1.000	0.565	1.16	0.26	1.13	0.17	1.09	0.21	0.386	0.895	0.501
ALT	45.88	62.94	29.27	24.14	29.82	19.86	0.103	0.179	0.396	28.67	22.11	27.06	28.55	22.40	14.56	0.566	0.170	0.279
AST	42.06	65.17	24.68	11.18	22.17	8.36	0.193	0.224	0.260	22.99	8.55	26.38	18.63	21.25	6.50	0.583	0.365	0.354
ALP	235.65	75.18	221.82	73.43	209.45	46.17	0.179	0.303	0.508	232.83	102.63	224.38	91.54	211.14	59.95	0.640	0.203	0.855

ALP, Alkaline phosphatase; ALT ,Alanine transaminase; AST, Aspartate transferase; BMI ,Body mass index; HbA1c, Glycated hemoglobin; HDL, High density lipoprotein; LDL, Low density lipoprotein.

**Table 3a:** Patients (n=17/69, 24.6%) with at least a 5% reduction in BMI in liraglutide group (T1-T3)*.

Start BMI (kg/m^2^)	End BMI (kg/m^2^)	Percentage Loss (%)
38.27	30.00	21.61%
32.00	26.50	17.19%
39.18	35.98	8.17%
30.80	27.40	11.04%
35.76	33.30	6.88%
41.43	39.22	5.33%
34.43	31.63	8.13%
38.26	35.10	8.26%
35.00	32.00	8.57%
35.00	31.00	11.43%
41.35	39.00	5.68%
34.20	31.20	8.77%
37.70	35.14	6.79%
37.70	32.05	14.99%
43.70	39.40	9.84%
38.89	34.30	11.80%
33.48	31.60	5.62%

*BMI reduction: 6/17 (35.3%) patients had more than 10% reduction.

**Table 4A:** Patients (n=10/69, 14.5%) with 5-10% reduction in weight in liraglutide group (T1-T3)*.

Start Weight (kg)	End Weight (kg)	Percentage Loss (%)
90.4	74.6	17.48%
120.0	110.2	8.17%
73.2	65.7	10.25%
83.7	79.5	5.02%
109.8	104.2	5.10%
80.6	76.0	5.71%
97.1	91.4	5.87%
89.0	83.0	6.74%
80.0	74.0	7.50%
97.2	92.0	5.35%

*Weight reduction: 8/10 (80%) patients had between 5 to 10% reduction; and 2 (20%) patients had more than 10% reduction.

**Table 3B:** Patients (n=11/69, 15.9%) with at least a 5% reduction in their BMI in lifestyle group (T1-T3)*.

Start BMI (kg/m^2^)	End BMI (kg/m^2^)	Percentage Loss (%)
41.00	32.00	21.95%
33.50	31.20	6.87%
32.25	30.26	6.17%
35.00	21.10	39.71%
40.50	32.60	19.51%
36.00	32.30	10.28%
32.69	24.41	25.33%
32.37	27.79	14.15%
35.82	28.77	19.68%
38.17	35.00	8.30%
44.30	33.10	25.28%

*BMI reduction: 2/11 (18%) patients had between 5 to 10% reduction; and 9/11 (81.8%) patients had more than 10% reduction.

**Table 4b:** Patients (n=11/69, 15.9%) with at least a 5% reduction in their weight in Lifestyle group (T1-T3)*.

Start Weight (kg)	End Weight (kg)	Percentage Loss (%)
102.0	80.0	21.57%
70.0	43.0	38.57%
159.0	105.0	33.96%
97.2	85.9	11.63%
98.0	87.0	11.22%
81.3	72.2	11.19%
91.0	77.5	14.84%
90.1	77.2	14.32%
91.7	87.0	5.13%
108.0	77.0	28.70%
76.9	49.0	36.28%

*Weight reduction: 1/11 (9%) patient had between 5 to 10% reduction; and 10 (91%) patients had more than 10% reduction.

**Figure 1:**
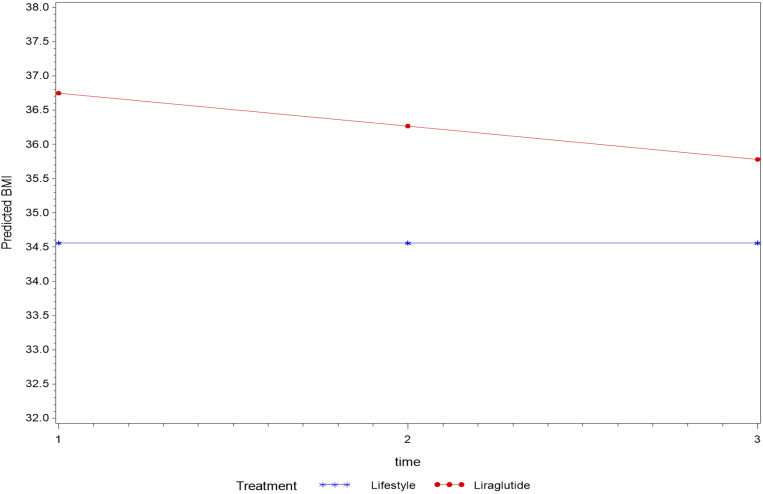
The average change of BMI (kg/m^2^) measurements overtime based on a linear mixed model- P=0.089 for the interaction between time and treatment, average BMI (kg/m^2^) differed among treatment groups (p=0.018).

Overall, treatment status had no effect on the average change in BMI levels over the entire study period using mixed model analysis (p=0.089). However, the average BMI measure was significantly higher in the liraglutide group (p=0.018) compared to the lifestyle group. Moreover, BMI measured over time within LiraglG group indicated an average decline of 0.48 kg/m2 during each measurement period (p=0.003) (**Figure 1**).

In an analysis that compared BMI measures at follow-up to the baseline measure by treatment status revealed a significant interaction effect (p=0.027) with BMI measured at the first follow-up (T2) indicating a possible effect of treatment until first follow-up that was not sustained thereafter.

Although regression analysis indicated no effect of treatment status on changes in average HbA1c levels over time (P=0.219) among the treatment groups, an average decline of 0.13% in HbA1c levels was observed during each follow-up period in patients treated with liraglutide (p=0.004). This drop was mainly observed in the female subgroup of the LiraglG cohort.

**Figure 2:**
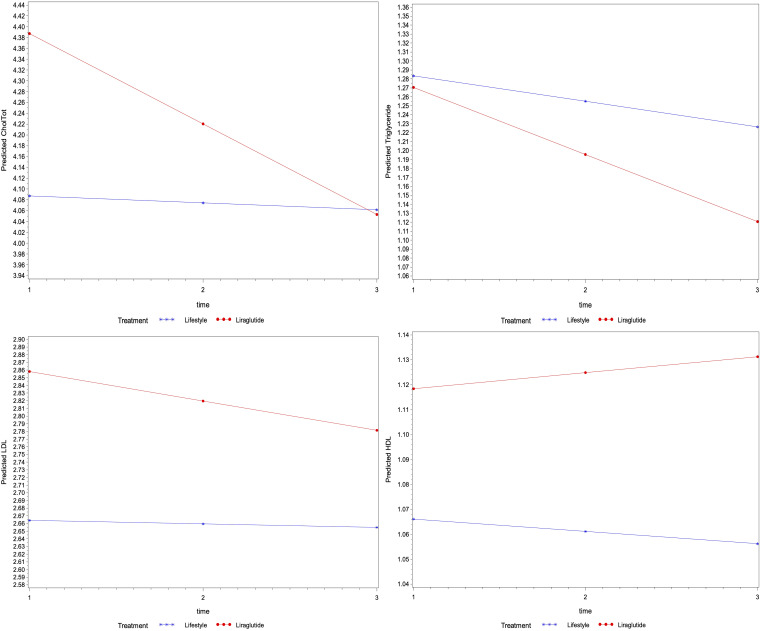
The average change of metabolic markers for lipids (mmol/l of total cholesterol, triglycerides, LDL and HDL) overtime based on their linear mixed models- P-values were 0.086, 0.682, 0.656 and 0.649 for the interaction between time and treatment for total cholesterol, triglycerides, LDL and HDL levels, respectively), the average levels of total cholesterol were different among treatment groups (p=0.049), whereas the level of triglycerides (p=0.899) LDL (p=0.292) and HDL (p=0.509) were not different.

**Figure 3:**
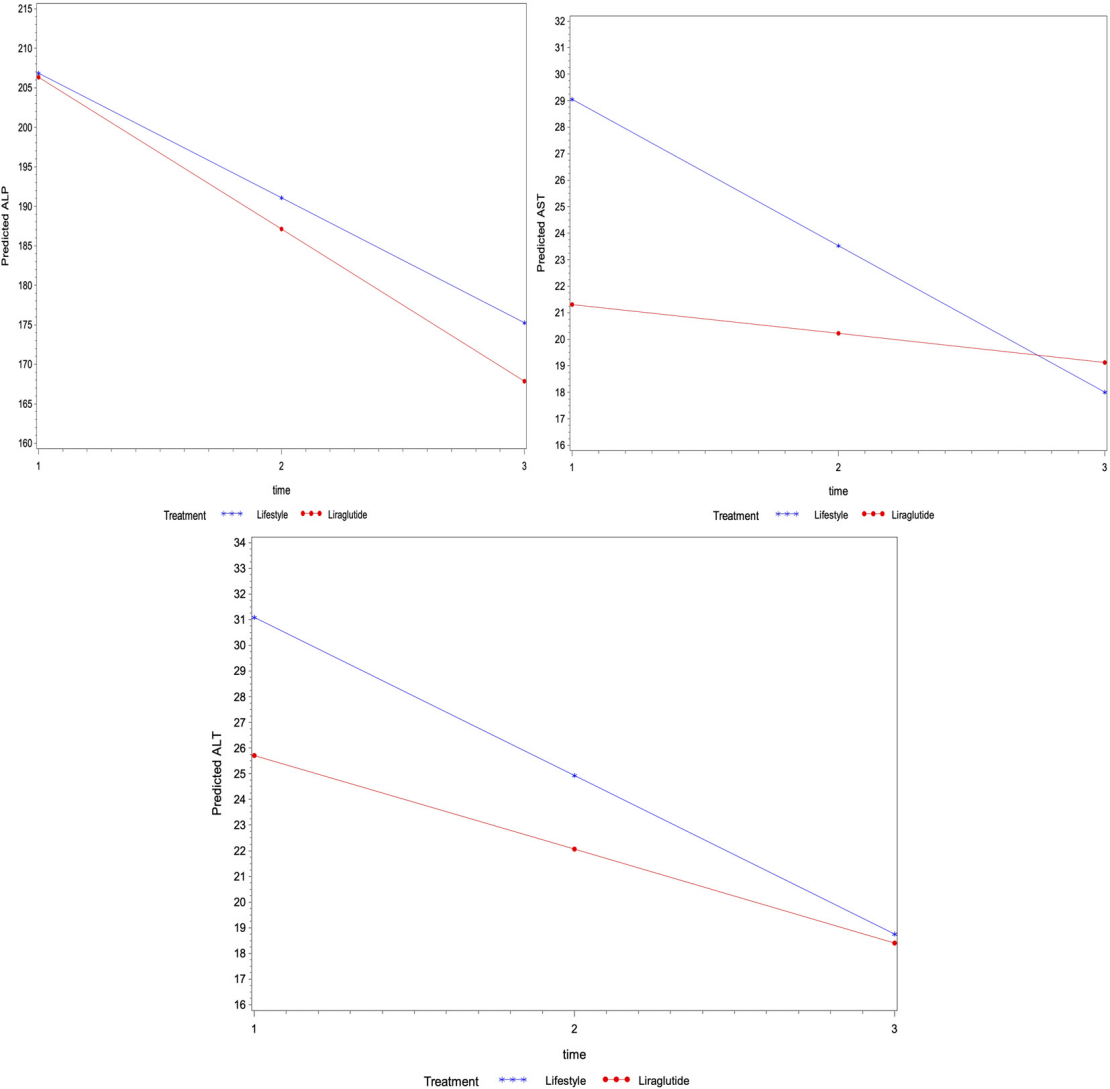
The average change of metabolic markers for liver function (U/L of ALP, ALT and AST) overtime based on linear mixed models – P-values were 0.671, 0.509 and 0.221 for the interaction between time and treatment for ALP, ALT and AST levels, respectively), the average levels of ALP (p=0.898) ALT (p=0.355) and AST (p=0.123) were not different among treatment groups.

Similarly, the regression analysis indicated no interaction effect of treatment on the average change in other biomarkers for lipid profile (i.e., total cholesterol, triglycerides, LDL and HDL) and liver function (i.e., ALT, AST and ALP) over time (p>0.05) (**Figures 2**, **3**). However, an average decline of 0.17 mmol/l in total cholesterol level (p=0.015), and 19.2 U/L in ALP level (p= 0.002) were observed over subsequent measurement periods in patients treated with liraglutide.

## Discussion

The findings from our study indicate that liraglutide, when used alongside lifestyle modifications, significantly impacts BMI reduction and leads to improvements in some metabolic markers in obese adolescents, especially in the female subgroup. The observed overall reduction in BMI in the LiraglG aligns with previous studies demonstrating the efficacy of GLP-1 receptor agonists in managing obesity. This reduction, although modest, is significant and highlights the potential of liraglutide as an effective adjunct therapy in obesity management for adolescents. Generally, integrating liraglutide with comprehensive lifestyle interventions not only enhances the efficacy of weight management strategies but also promotes sustained positive outcomes through the synergistic effects of pharmacological and behavioral approaches.

A noteworthy observation from our study is that the beneficial effects of liraglutide on BMI are more pronounced during the first 3 to 6 months of therapy. Although further weight loss after 6 months of liraglutide treatment was limited, the initial weight loss was maintained after 9 to 12 months of treatment. This was further supported by our finding in the subgroup analysis of patients who had significant 5-10% weight/BMI reduction in both study groups, where a better clinical effect was clearly observed with sustainable intervention in the LifeSG compared to lesser effect in these patients in the LiraglG. Some patients in the LiraglG had possibly lost their motivation and self-commitment during the study period regarding the lifestyle modifications and totally relied on the medication’s effect. These findings suggests that while liraglutide can initially boost BMI reduction, its long-term efficacy may diminish without continuous support from lifestyle interventions. Unfortunately, pediatric data on the benefit of long-term use of liraglutide on weight loss and BMI reduction for more than one year are limited. Additionally, there are concerns regarding rebound weight gain after discontinuing GLP-1 agonists (14). This further underscores the importance of adopting healthy lifestyle habits to maintain reduced weight after discontinuing liraglutide.

Adult studies have demonstrated the efficacy of liraglutide in promoting weight loss in overweight and obese adults, with or without type 2 diabetes showing more significant results compared to metformin or placebo (15). Consistent with our results, adult data demonstrate that the majority of weight loss occurs during the first year of liraglutide treatment, and this weight loss is maintained over a two-year-follow-up period, with improvement in obesity-associated metabolic markers (16). Adult overweight and obese patients with type 2 diabetes also demonstrated significant weight-loss with short-term liraglutide treatment, accompanied by improvements in HbA1c and insulin resistance; however, long-term benefits require sustained treatment and lifestyle modifications (15).

In the pediatric population, liraglutide has been proven to have a beneficial effect on reducing BMI scores in obese youth aged 12 years and older when compared with placebo in the setting of a randomized controlled clinical trial and is FDA-approved for this indication in this age group (12). Regarding predictors of liraglutide’s weight reducing effect, response to liraglutide is not affected by age, gender, ethnicity/race, pubertal status, glycemia or obesity class (17). Additionally, a more recent randomized clinical trial that included obese children between the ages of 6 and 12 years was conducted to study the weight-reducing effect and safety of liraglutide in this age group compared to placebo (18). There was a significantly greater reduction in BMI in the group treated with liraglutide and lifestyle intervention compared to those in the placebo group (18). In children with syndromic obesity, who were not included in our study, liraglutide also has a positive impact. Liraglutide seems to improve hyperphagia in adolescents with Prader-Willi Syndrome, as those treated with liraglutide had lower hyperphagia scores compared to those who did not receive treatment (19). Liraglutide may also be an option for adolescents with persistent obesity after sleeve gastrectomy, as evidenced by an open-label trial that included 34 participants (20). Treatment with liraglutide resulted in a 4.3% reduction in BMI as well as improvements in fasting glucose and HbA1c (20).

Our results of substantial reductions in total cholesterol and LDL levels are consistent with those reported recently by other investigators, who noted significant metabolic benefits of liraglutide, with or without combination with metformin, in reducing BMI and HbA1c, and improving the lipid profile (21, 22). This further highlights the role of liraglutide in improving cardiovascular risk factors associated with obesity. However, the lack of sustained improvement in the HbA1c and some other biomarkers in our study suggests that liraglutide alone, without sustained lifestyle interventions, may not be sufficient for comprehensive metabolic control over extended periods.

The findings from our study indicate that liraglutide treatment combined with lifestyle modifications did not result in a sustained and significant improvement in the HbA1c compared to its prolonged effect on other metabolic biomarkers such as total cholesterol and ALP levels. These results contrast the findings from other similar studies that demonstrated significant glycemic control and sustained improvement in HbA1c levels with liraglutide treatment in pediatric populations who had type 2 diabetes (13, 23, 24). Recent reviews highlighted randomized controlled trials that provided evidence of the effectiveness of liraglutide in reducing HbA1c in children and adolescents with obesity and type 2 diabetes (23, 24). Similarly, a recent meta-analysis that confirmed the safety and efficacy of GLP-1 receptor agonists, including liraglutide, in improving glycemic control among obese pediatric patients and/or those with type 2 diabetes (13). A possible explanation for our finding is that the obese children in our study did not have type 2 diabetes but rather had HbA1c levels within the pre-diabetes range. Although, patients in the LiraglG, especially the female subgroup, experienced a decline of 0.13% in A1c over time, this was likely not clinically meaningful except in those who were in the pre-diabetes range of A1c (≥ 5.8%). The discrepancy in findings between our study and other published data could also be due to differences in study designs, duration of follow-up, patients’ adherence to treatment and lifestyle modifications, or the baseline characteristics of the study populations. Overall, the lack of sustained glycemic control in our study suggests that liraglutide alone may not be sufficient for comprehensive metabolic control over extended periods, highlighting the need for continuous lifestyle interventions and possibly coaching and monitoring the obese Saudi adolescents to achieve long-term metabolic control.

A limitation of our study is its retrospective design, which limits the ability to establish causality between liraglutide use and the observed outcomes. Additionally, the study was conducted at a single centre in Riyadh, which may limit the generalizability of its findings to other populations. Unfortunately, we did not have sufficient retrospective data on insulin sensitivity in our cohort; however, this would be useful additional data to include in further prospective analyses of these patients. Finally, the relatively short duration of follow-up may not fully capture the long-term effects and potential adverse effects of liraglutide treatment.

In conclusion, liraglutide can be a valuable addition to lifestyle interventions for obese adolescents, especially in the early months of treatment. The significant reductions in BMI and certain metabolic markers indicate that liraglutide could be an effective strategy to enhance the efficacy of lifestyle modifications, particularly in patients who are resistant to lifestyle changes alone. However, the variability in the sustainability of these effects underscores the importance of continuous lifestyle interventions to maintain these benefits over the long term.

## Data Availability

The original contributions presented in the study are included in the article/supplementary material. Further inquiries can be directed to the corresponding author.
